# Triaging Shakespeare

**DOI:** 10.7554/eLife.07157

**Published:** 2015-03-17

**Authors:** Indira M Raman

**Affiliations:** Department of Neurobiology, Northwestern University, Evanston, United Statesi-raman@northwestern.edu

**Keywords:** Living science, science policy, NIH, funding

## Abstract

What if every creative endeavor had to go through Peer Review? **Indira M Raman** considers the possibility.

**To:** Ben Jonson **bjonson@newgate.gov**

**From:** Wm. Shakespeare **shakes@globe.org**

**Subject:** Triaged!

**Date:** February 4, 1599 8:03:03 AM GMT

Dear Ben,

I just got the reviews back on my grant proposal for *Hamlet,* the new play I was telling you about. I applied to the National Institutes of Health in America this time—the RFA said they're interested in creative new ideas and all that rot but they're just as conservative as here in England. It wasn't even discussed! I'll wager it was those scoundrels Beaumont and Fletcher that sank it. I could self-publish again, but, ’zounds, I could use some cash for a couple of new swords for the final scene. Of course, if I were a swashbuckler like Raleigh, I'd just get funded by the Queen. Can't anyone see that it's not globetrotting, but the Globe, that teaches people how to *think*? Anyway, I'd appreciate your input on what to do next. (Do you think Cervantes would be up for an HFSP?) I'm attaching the Significance and Innovation sections of the proposal, so you can get a feel for the idea, followed by the critique.

Very truly yours,

Will

P.S. I'm not as glum as I sound. I'll get this thing written or my name isn't William Shakespeare.

P.P.S. Glad you'll be out soon.

## Project Title: “The Tragedy of Hamlet, Prince of Denmark”

### Significance

Understanding the human condition is arguably one of the most pressing issues facing mankind. Specifically, elucidating the sensory experience, internal integration, and motor consequences of joy, sorrow, rage, and despair is critical for knowing what it means to be alive, conscious, and self-aware. Previous studies in these areas have given rise to multiple great works of literature, both tragic (cf. Aeschylus 472 BCE, Sophocles 442 BCE, Euripides 438 BCE) and comic (Aristophanes 425 BCE). Nevertheless, the human condition is still poorly understood. The present proposal will make use of tragedy to explore this phenomenon, focusing on betrayal and vengeance. Both factors will be examined through a protagonist, ‘Hamlet,’ whose hesitation to act is predicted to influence the behavior, and ultimately cause the death, of multiple characters. The work therefore has the potential to unify disease states as diverse as madness, sword wounds, and poisonings as deriving from a single underlying mechanism, indecision.

### Innovation

In Act III of *Hamlet*, I will implement the novel technique of a play-within-a-play. This new device will shift current paradigms of playwriting, in which player and spectator have (to my knowledge) never before been one and the same man. Moreover, by distinguishing the reflective Hamlet from the emotional performer, I will provide the spectators with new insights into the nature of Hamlet's problem. As such, this method has advantages over current practices, which require the spectator to make indirect inferences about character motivations from the complete performance, which can often require two or more hours. An additional advantage is that the utility of this method will likely motivate other playwrights to adopt the same approach. Most importantly, it will offer a topic of analysis for literary critics, i.e., those who generate no creative arts but simply evaluate the creativity of others, that will likely keep them busy for at least four centuries.

## SUMMARY STATEMENT

**(Privileged Communication)**

**Release Date: 2/1/1599**

***Application Number:* 1 R01 NS00002**

**Principal Investigator**

**SHAKESPEARE, WILLIAM**

***Project Title:* “The Tragedy of Hamlet, Prince of Denmark”**

**SRG Action: Impact/Priority Score: 52 Percentile: UN**

### Critique 1

#### Significance      5

##### Strengths:

‐      Understanding the human condition is potentially of high impact. It should be pointed out, however, that previous investigators have tried and failed, so this should be seen as a high-risk-high-gain endeavor.
‐      Relating indecision to pathologies may help identify novel therapeutic targets.

##### Weaknesses:

‐      The proposal is essentially descriptive. Significance would be enhanced if the applicant cloned the gene for indecision. The play could then be repeated with an indecision-knockout Hamlet and the relevance of indecision to madness, etc could be more directly assessed.
‐      While betrayal and vengeance are undoubtedly important elements of the human experience, the applicant neglects love and suffering.

#### Approach      6

##### Strengths:

‐      Excellent consideration of null and alternative hypotheses in proposed soliloquy of Act III.

##### Weaknesses:

‐      Preliminary data are not compelling, esp. regarding evidence for the applicant's contention that King Hamlet is really dead. Two issues are problematic. (1) Death of Hamlet, Sr., is assumed because poison was poured in his ear. This is a non-standard approach and is not sufficiently justified. What kind of poison was it and at what concentration was it used? Does this poison cross the blood brain barrier? What is its mode of pharmacological action? Why was poison excluded from the contralateral ear? Without further information about mechanism, death cannot be assumed. Note also that the poison was apparently not followed by thoracic puncture, although this issue is admittedly handled more effectively in Act V. (See also comments under ‘Protections for Human Subjects’). (2) The appearance of the King at the beginning of Act I to tell of his death is not consistent with his actual death before the play begins. The (likely) possibility of a flawed premise therefore raises concerns about the remaining Acts.
‐      Outcomes are not adequately discussed. For instance, in Act III, when Hamlet stabs the curtain in Gertrude's closet, it is assumed that Polonius will be killed. A plausible alternative, however, is that Hamlet will kill a rat, as he himself hypothesizes. This possibility should be considered explicitly. If it indeed turns out to be a rat, the Vertebrate Animals section should be completed.
‐      Likewise, in Act V, if Laertes recovers from his poisoned wound, will Fortinbras still be appointed King? Some discussion is necessary.
‐      Biohazards are not explicitly considered, e.g., in Act V, will surgical gloves (latex or nitrile) be used to handle Yorick's skull; how will carcasses be disposed of, etc. (N.B. The proposed concealment of Polonius’ body in Act IV is unacceptable).

#### Innovation      5

##### Strengths:

‐      The use of terminology is innovative, e.g., naming a Dane Hamlet.
‐      The work contains cutting-edge techniques.

##### Weaknesses:

‐      The technique of a play-within-a-play is not novel. A brief literature search indicates that it was originally used more than a decade ago, by Thomas Kyd in 1587. The author himself has used it twice before, in A Midsummer Night's Dream and Love's Labours Lost, only a few years ago. Thus, use of this method is likely to be of only incremental value.

#### Investigator      4

##### Strengths:

‐      The applicant has a fairly good track record, with 21 plays performed in the last ten years (11 in the last project period).
‐      The applicant is well-versed in iambic pentameter.

##### Weaknesses:

‐      There is no evidence that the applicant has previous experience writing about 13^th^ century Denmark (or even general familiarity with the subject). Enthusiasm is dampened by the absence of supporting letters from colleagues with expertise in this area.
‐      Despite the applicant's high productivity, it is not clear that such an ambitious project will be completed within the project period without recruitment of additional collaborators (e.g., Francis Bacon, Christopher Marlowe, or Edward de Vere, 17th Earl of Oxford).

#### Environment      2

##### Strengths:

‐      The Globe Theatre is an outstanding environment.
‐      Elizabethan England is rapidly becoming a significant world power.

##### Weaknesses:

None.

